# Potent prophylactic and therapeutic efficacy of recombinant human ACE2-Fc against SARS-CoV-2 infection in vivo

**DOI:** 10.1038/s41421-021-00302-0

**Published:** 2021-08-12

**Authors:** Zhaoyong Zhang, Eric Zeng, Lu Zhang, Weiming Wang, Yingkang Jin, Jiye Sun, Shuxiang Huang, Wenguang Yin, Jun Dai, Zhen Zhuang, Zhao Chen, Jing Sun, Airu Zhu, Fang Li, Weitao Cao, Xiaobo Li, Yongxia Shi, Mian Gan, Shengnan Zhang, Peilan Wei, Jicheng Huang, Nanshan Zhong, Guocai Zhong, Jingxian Zhao, Yanqun Wang, Weihui Shao, Jincun Zhao

**Affiliations:** 1 State Key Laboratory of Respiratory Disease, National Clinical Research Center for Respiratory Disease, Guangzhou Institute of Respiratory Health, the First Affiliated Hospital of Guangzhou Medical University, Guangzhou, Guangdong China; 2 Nanjing Legend Biotech Co., Ltd, Nanjing, Jiangsu China; 3 Guangzhou Customs District Technology Center, Guangzhou, Guangdong China; 4 Nanjing GenScript Biotech Co., Ltd, NanJing, Jiangsu China; 5 Pediatric Pulmonary Department, Guangzhou Women and Children’s Medical Center, Guangzhou Medical University, Guangzhou, Guangdong China; 6 Shenzhen Bay Laboratory, Shenzhen, Guangdong China; 7 School of Chemical Biology and Biotechnology, Peking University Shenzhen Graduate School, Shenzhen, Guangdong China; 8 Institute of Infectious disease, Guangzhou Eighth People’s Hospital of Guangzhou Medical University, Guangzhou, Guangdong China; 9 Guangzhou laboratory, Bio-island, Guangzhou, Guangdong China

**Keywords:** Mechanisms of disease, Immunology

## Abstract

The current COVID-19 pandemic, caused by SARS-CoV-2, poses a serious public health threat. Effective therapeutic and prophylactic treatments are urgently needed. Angiotensin-converting enzyme 2 (ACE2) is a functional receptor for SARS-CoV-2, which binds to the receptor binding domain (RBD) of SARS-CoV-2 spike protein. Here, we developed recombinant human ACE2-Fc fusion protein (hACE2-Fc) and a hACE2-Fc mutant with reduced catalytic activity. hACE2-Fc and the hACE2-Fc mutant both efficiently blocked entry of SARS-CoV-2, SARS-CoV, and HCoV-NL63 into hACE2-expressing cells and inhibited SARS-CoV-2 S protein-mediated cell–cell fusion. hACE2-Fc also neutralized various SARS-CoV-2 strains with enhanced infectivity including D614G and V367F mutations, as well as the emerging SARS-CoV-2 variants, B.1.1.7 (Alpha), B.1.351 (Beta), B.1.617.1 (Kappa), and B.1.617.2 (Delta), demonstrating its potent and broad-spectrum antiviral effects. In addition, hACE2-Fc proteins protected HBE from SARS-CoV-2 infection. Unlike RBD-targeting neutralizing antibodies, hACE2-Fc treatment did not induce the development of escape mutants. Furthermore, both prophylactic and therapeutic hACE2-Fc treatments effectively protected mice from SARS-CoV-2 infection, as determined by reduced viral replication, weight loss, histological changes, and inflammation in the lungs. The protection provided by hACE2 showed obvious dose-dependent efficacy in vivo. Pharmacokinetic data indicated that hACE2-Fc has a relative long half-life in vivo compared to soluble ACE2, which makes it an excellent candidate for prophylaxis and therapy for COVID-19 as well as for SARS-CoV and HCoV-NL63 infections.

## Introduction

The Coronavirus Disease 2019 (COVID-19) pandemic, which is caused by severe acute respiratory syndrome coronavirus 2 (SARS-CoV-2), poses a significant public health threat^1^. As of June 20, 2021, over 178 million confirmed cases were reported, with more than 3.8 million deaths^2^. With global effort, more than 2 billion vaccine doses have been administrated worldwide. Typical symptoms of SARS-CoV-2 infection included fever, cough, fatigue, shortness of breath. Severely ill patients developed complications including pneumonia, acute respiratory distress syndrome (ARDS), and multi-organ failure^3–5^. The pandemic has caused global social disruption and massive economic losses. However, there were currently no effective drugs approved for human use to prevent or treat COVID-19, except for remdesivir and two neutralizing antibody cocktails (Eli Lilly and Regeneron), although they are not so efficient to treat patients with severe disease^6,7^. Recently, multiple SARS-CoV-2 Variants of Concern (VOCs) were reported to spread globally in a short period of time, such as B.1.1.7 (known as 20I/S: 501Y.V1 or Alpha strain) first emerged in the United Kingdom (UK), B.1.351 (known as 20H/S: 501Y.V2 or Beta strain) first emerged in South Africa, P.1 (known as 20 J/S: 501Y.V3 or Gamma strain) first emerged in Brazil, and B.1.617.2 (known as 21 A/S: 478 K or Delta strain) first emerged in India, some of which showed reduced sensitivity to some therapeutic antibodies isolated from convalescents^8–13^.

SARS-CoV-2 entry depends on the binding of spike protein receptor binding domain (RBD) to its receptor, angiotensin-converting enzyme II (ACE2)^1,14^, the same cellular receptor utilized by SARS-CoV^15,16^. SARS-CoV-2 RBD exhibited higher binding affinity to ACE2, compared to its counterpart in SARS-CoV^15,17^. Thus, chemicals, proteins and antibodies that block the binding of ACE2 to RBD could potentially be used for COVID-19 prevention and control^18–22^. Many potential therapies for COVID-19 have been reported recently, including neutralizing antibody, convalescent plasma from patients recovered from COVID-19, repurposed drugs with anti-SARS-CoV-2 potentials, etc., which need further investigation for their safety and efficacy^7,19,23–28^.

Human recombinant soluble ACE2 has been shown to inhibit SARS-CoV^29^ and SARS-CoV-2 infection in vitro^30,31^. While murine recombinant soluble ACE2 did not significantly affect SARS-CoV-2 infections, highlighting the specificity of human ACE2 (hACE2) in blocking SARS-CoV-2 entry^31^. Recombinant hACE2 can not only block the binding of spike protein of SARS-CoV to ACE2, but also regulate the renin-angiotensin system to reduce the histological damage caused by SARS^32^. A set of affinity-optimized ACE2 variants with reduced ACE2 catalytic activity-mutant were developed, which potently blocked SARS-CoV-2 infection in vitro^33,34^. Of note, soluble recombinant human ACE2 has a short half-life and is quickly cleared in vivo, while the fusion protein of hACE2 with immunoglobulin crystallizable fragment (Fc; hACE2-Fc), which showed a relatively long half-life without losing its biological function in hypertension treatment^35^.

Most of the efficacy studies described above were performed in vitro. The protective effect of hACE2-Fc against SARS-CoV-2 in vivo remains unclear, which significantly hinders its use in human clinically. Recently, we developed a novel mouse model for SARS-CoV-2 infection using adenovirus hACE2 transduction technology, which is broadly useful for the evaluation of vaccines, antibodies, and drugs in vivo^5^. Here, we comprehensively investigated the potential protective effect of recombinant hACE2-Fc, which not only potently neutralized SARS-CoV-2 in vitro but also showed an effective in vivo protective effect in SARS-CoV-2-infected mice. In addition, we also demonstrated that hACE2-Fc can efficiently neutralize SARS-CoV and HCoV-NL63 in vitro, which share the common cell receptor hACE2. These data suggested that hACE2-Fc is an excellent candidate for prophylaxis or treatment of COVID-19.

## Results

### hACE2 fused with Fc showed high binding affinity to SARS-CoV-2 Spike and RBD

**Fig. 1:**
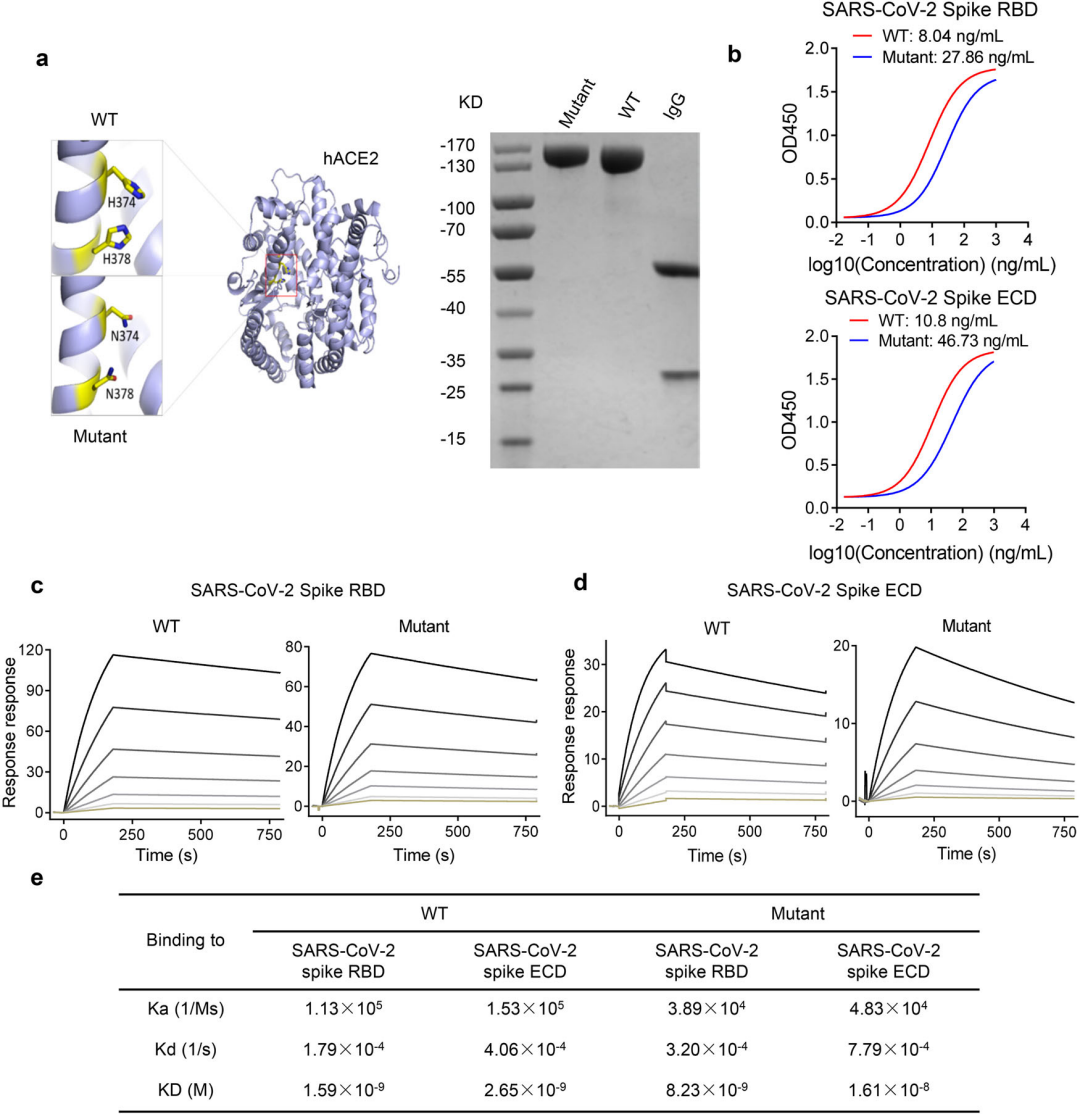
Expression, binding, and affinity of hACE2-Fc. **a** Diagrams of protein structure model and SDS-PAGE analysis of hACE2-Fc WT and hACE2-Fc mutant. **b** Binding specificity of hACE2-Fc WT and hACE2-Fc mutant. 96-well plate was coated with protein SARS-CoV-2 RBD and S, respectively (0.5 μg/mL), and serially diluted hACE2-Fc was added. HRP-labeled mouse anti-human IgG-Fc antibody was used as ELISA secondary antibody. Three independent experiments were performed, of which one representative result was shown. **c, d** hACE2-Fc show a potent binding to SARS-CoV-2 Spike ECD and RBD in Biacore assay. SARS-CoV-2 Spike ECD and RBD was immobilized on the chip surface, followed by injecting diluted analytes (hACE2-FC WT: 1.14 nM, 2.28 nM, 4.56 nM, 9.12 nM, 18.25 nM, 36.5 nM, 73 nM; hACE2-Fc mutant: 1.5625 nM, 3.125 nM, 6.25 nM, 12.5 nM, 25 nM, 50 nM, 100 nM, 200 nM) as association phase and injecting running buffer as dissociation phase (Biacore 8 K, GE Healthcare). All the data were processed using the Biacore 8 K Evaluation software version 1.1. **e** Summary of SPR assay. Association constant (Ka), dissociation constant (Kd) and equilibrium dissociation constant (KD) of hACE2-Fc (WT and mutant) against Spike ECD and RBD were calculated by Biacore 8 K. KD calculated as Ka/Kd, a smaller value represents a more potent binding capacity.

Recombinant hACE2-Fc protein was generated by fusing hACE2 extracellular domain to the N terminal of human IgG-Fc region (IgG-Fc fragment). Corresponding hACE2-Fc mutant protein with catalytic inactivation mutations (H374N and H378N mutations) was also generated as shown in Fig. 1a. Mutant hACE2-Fc was generated by replacing the ACE2 active-site histidines 374 and 378 with asparagines to eliminate catalytic motif, which inactivates ACE2’s catalytic activity but not affects its binding capacity^29,36^. Both proteins were produced in transiently transfected Expi293 cells and purified by protein A magnetic beads. Proteins were identified on a reduced SDS-PAGE gel with molecular weights at ~130 kilodalton (kDa), which was consistent with the predicted molecular weight of their monomers (Fig. 1a). The binding specificity of hACE2-Fc to SARS-CoV-2 RBD and spike ectodomain (ECD) was confirmed by enzyme linked immunosorbent assay (ELISA). Both hACE2-Fc wild-type (WT) and hACE2-Fc mutant efficiently bound to SARS-CoV-2 RBD and spike ECD (EC_50_ (value of half maximal effective concentration), 8.04 ng/mL and 27.86 ng/mL for RBD, 10.8 ng/mL and 46.73 ng/mL for spike ECD, respectively) (Fig. 1b). Of note, hACE2-Fc WT exhibited higher binding efficacies than the hACE2-Fc mutant, as assessed using Surface Plasmon Resonance (SPR). Both hACE2-Fc WT and the hACE2-Fc mutant displayed nanomolar affinity to SARS-CoV-2 RBD and spike ECD (Fig. 1c–e) (equilibrium dissociation constant (KD), 1.59 nM and 8.23 nM to RBD, respectively; 2.65 nM and 16.1 nM to ECD, respectively], which were comparable to some of the most potent SARS-CoV-2-specific neutralizing monoclonal antibodies isolated from COVID-19 convalescents^18,19,37^.

### hACE2-Fc potently neutralized SARS-CoV-2, SARS-CoV, and HCoV-NL63 in vitro

**Fig. 2:**
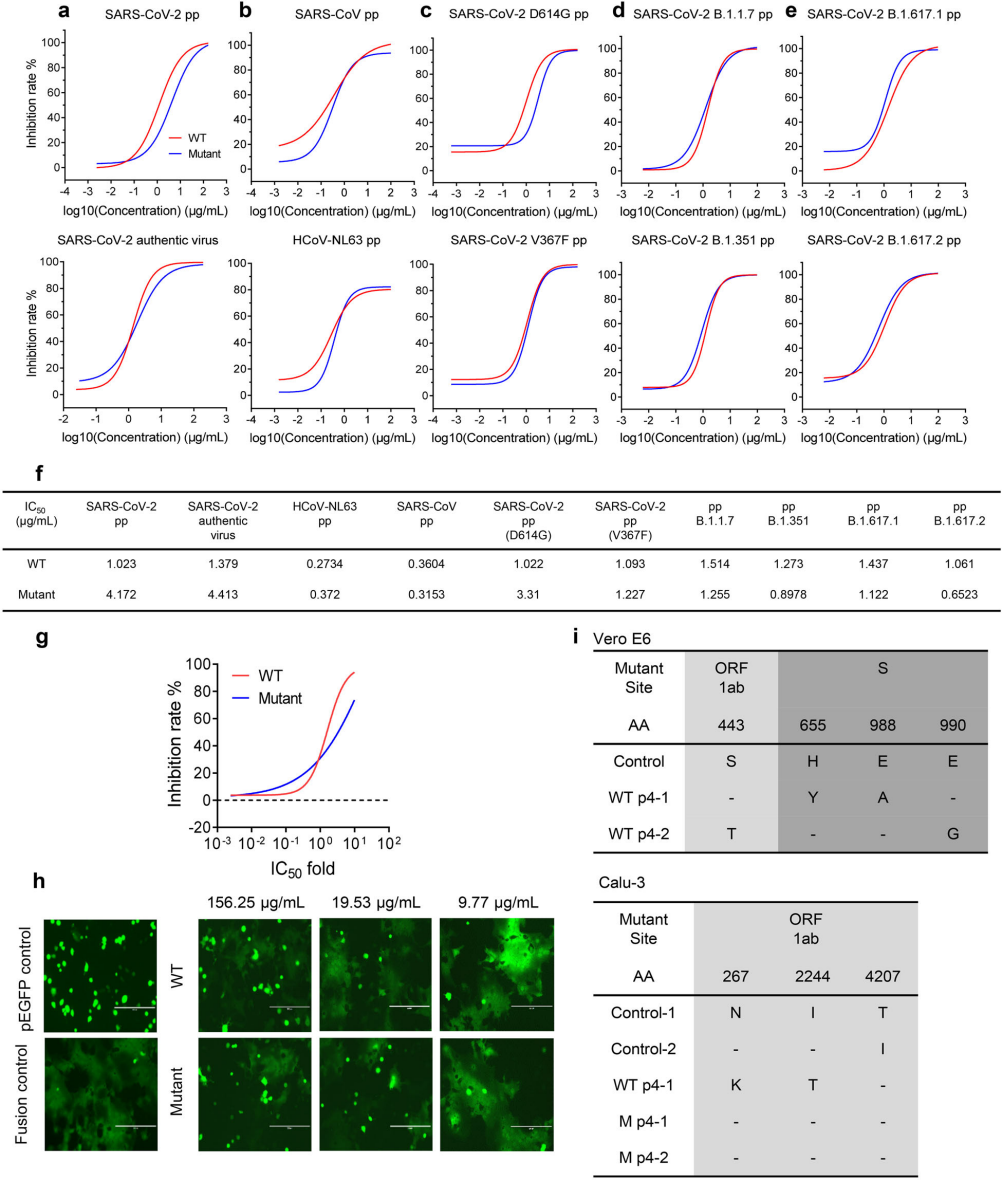
hACE2-Fc potently neutralized SARS-CoV-2, SARS-CoV, and HCoV-NL63 in vitro, inhibited SARS-CoV-2 S protein-mediated cell–cell fusion and did not induce escape mutants. **a** hACE2-Fc potently neutralized SARS-CoV-2 pseudotyped virus (pp) and authentic SARS-CoV-2 in vitro. HEK293-ACE2 cells or Vero E6 cells were infected with SARS-CoV-2 pp or SARS-CoV-2 authentic virus in the presence of serially diluted hACE2-Fc, respectively. Luciferase activity in pseudovirus-infected 293-ACE2 cells was detected 40 h post infection and inhibition rates were calculated. Vero E6 cells infected with SARS-CoV-2 authentic virus were fixed 1 day after infection. Focus reduction neutralizing assay was performed as described in the experimental procedures. Date points are mean ± SEM of three biological replicates with two independent experiments. **b** HEK293-ACE2 cells or Huh7 cells were infected with SARS-CoV pp or HCoV-NL63 pp in the presence of serially diluted hACE2-Fc, respectively. Luciferase activity in pseudovirus-infected cells was detected 40 h post infection and inhibition rates were calculated. Date points are mean ± SEM of three biological replicates with two independent experiments. **c** hACE2-Fc potently neutralized SARS-CoV-2 pp mutant bearing D614G or V367F. Date points are mean ± SEM of three biological replicates with two independent experiments. **d** hACE2-Fc potently neutralized SARS-CoV-2 emerging B.1.1.7 and B.1.351 variants. Date points are mean ± SEM of three biological replicates with two independent experiments. **e** hACE2-Fc potently neutralized SARS-CoV-2 variants B.1.617.1 and B.1.617.2. Date points are mean ± SEM of three biological replicates with two independent experiments. **f** Summary of IC_50_ of neutralization assay. **g** hACE2-Fc WT and mutant did not promote authentic SARS-CoV-2 infection at a wide range of concentration. Date points are mean ± SEM of six biological replicates with two independent experiments. **h** hACE2-Fc inhibited SARS-CoV-2 S protein-mediated cell–cell fusion. HEK293T cells transfected with SARS-CoV-2 S and pEGFP were preincubated with serially diluted hACE2-Fc WT or mutant for 1 h at 37 °C. Cells were then mixed with Huh7 cells for membrane–membrane fusion. Cells transfected with pEGFP vector were used as negative control. After 24 h, the formation of syncytium was observed. Scale bars (white), 200 µm. Data are representative of three independent experiments. **i** Sequencing of hACE2-Fc escape mutant viruses. Four rounds of passage were performed in SARS-CoV-2-infected Vero E6 cells and Calu-3 cells in the presence of hACE2-Fc ranging from 0.4× to 8× IC_50_. Viral RNA was extracted and sequenced. Two independent passages of SARS-CoV-2 with/without hACE2-Fc WT were performed.

Neutralizing activity was assessed using both SARS-CoV-2 Spike pseudotyped (SARS-CoV-2 pp) and authentic virus neutralization assays. hACE2-Fc WT demonstrated strong neutralizing activity against SARS-CoV-2 (value of half maximal inhibitory concentration, IC_50_, 1.023 μg/mL for SARS-CoV-2 pp, 1.379 μg/mL for authentic virus), while hACE2-Fc mutant had modestly lower neutralizing activity (IC_50_, 4.172 μg/mL for SARS-CoV-2 pp, 4.413 μg/mL for SARS-CoV-2 authentic virus) (Fig. 2a), as compared with hACE2-Fc WT. HCoV-NL63 and SARS-CoV pp neutralization assays were also performed to examine the neutralization breadth of hACE2-Fc since both of these two viruses use ACE2 as their entry receptor^33,38,39^. Both hACE2-Fc WT and mutant displayed high neutralizing activity against HCoV-NL63 and SARS-CoV pp as shown in Fig. 2b. Considering that multiple variations of SARS-CoV-2 are prevalent around the world, six emerging SARS-CoV-2 pp were generated bearing the D614G mutation, V367F mutation and the mutations present in B.1.1.7 (Alpha strain), B.1.351 (Beta strain), B.1.617.2 (Delta strain), and B.1.617.1 (Kappa strain)^8,12,40,41^. Spike protein with the D614G mutation was considered as a key mutation to increase virus transmission and infectivity, and the V367F mutant exhibited significantly increased affinity to hACE2, while VOCs may increase virulence and reduce sensitivity to some neutralizing antibodies and convalescent sera at different extent^9,10,40–45^. As shown in Fig. 2c, hACE2-Fc WT and hACE2-Fc mutant showed comparable neutralizing activity against SARS-CoV-2 mutants bearing D614G, and was similar to the neutralizing activity found using WT SARS-CoV-2 pp. Similar inhibitory activity using WT and mutant hACE2-Fc was detected against SARS-CoV-2 mutant bearing V367F. Both hACE2-Fc WT and mutant also showed potent neutralization activity against B.1.1.7, B.1.351, B.1.617.2, and B.1.617.1, indicating the advantage of hACE2-Fc to avoid SARS-CoV-2 escape mutants from being neutralized (Fig. 2d, e). H374N and H378N mutants did not alter SARS-CoV-2 neutralizing capacity of hACE2-Fc. IC_50_s of WT and mutant hACE2-Fc were summarized in Fig. 2f. Unlike CD4, which inhibits HIV-1 infection at high concentration^46^, but promotes infection at low concentration^47^, both hACE2-Fc WT and mutant did not promote infection over a wide range of concentrations (hACE2-Fc WT, mutant: 0.002 × IC_50_–10 × IC_50_) (Fig. 2g).

### hACE2-Fc inhibited SARS-CoV-2 S protein-mediated cell–cell fusion

Further, in order to determine whether hACE2-Fc could inhibit spike protein-mediated cell–cell fusion as some neutralizing antibodies do^48^, HEK293T cells (effector cells) co-transfected with pcDNA3.1-SARS-CoV-2 Spike plasmid and pEGFP plasmid were preincubated with serially diluted hACE2-Fc WT or mutant for 1 h. HEK293T cells were then co-cultured with Huh7 cells (target cells), which have high hACE2 expression on cell surface. After 24 h cell–cell fusion between target cells and effector cells, evident syncytium could be observed under fluorescence microscopy (Fig. 2h). Effective reductions of syncytia were observed after hACE2-Fc WT and mutant treatment in a dose-dependent manner, indicating that both hACE2-Fc WT and mutant potently inhibit fusion (Fig. 2h).

### Serial passage did not generate escape mutants in the presence of hACE2-Fc

To investigate whether SARS-CoV-2 could escape neutralization by hACE2-Fc after serial passage in mammalian cells, in vitro selection of SARS-CoV-2 escape mutants under the pressure of hACE2-Fc was performed. Mutant sites in spike appear at the first or second round passage of SARS-CoV-2 incubated with single-potent SARS-CoV-2 neutralizing antibody^49^. Herein, SARS-CoV-2 was serially passaged with hACE2-Fc in Vero E6 cells, a cell line support ingrapid SARS-CoV-2 replication, and Calu-3 cells, a human lung adenoma cell line. At passage 4 (P4), no variants containing amino acid substitutions within the RBD was detected, indicating that unlike neutralizing monoclonal antibodies targeting a single epitope on RBD, hACE2-Fc covers the entire contact surface of ACE2 and RBD, and has the advantage of avoiding neutralization escape, which is related with the broad neutralizing activity of hACE2-Fc against VOCs (Fig. 2i).

### hACE2-Fc protected human bronchial epithelial (HBE) cells from SARS-CoV-2 infection

**Fig. 3:**
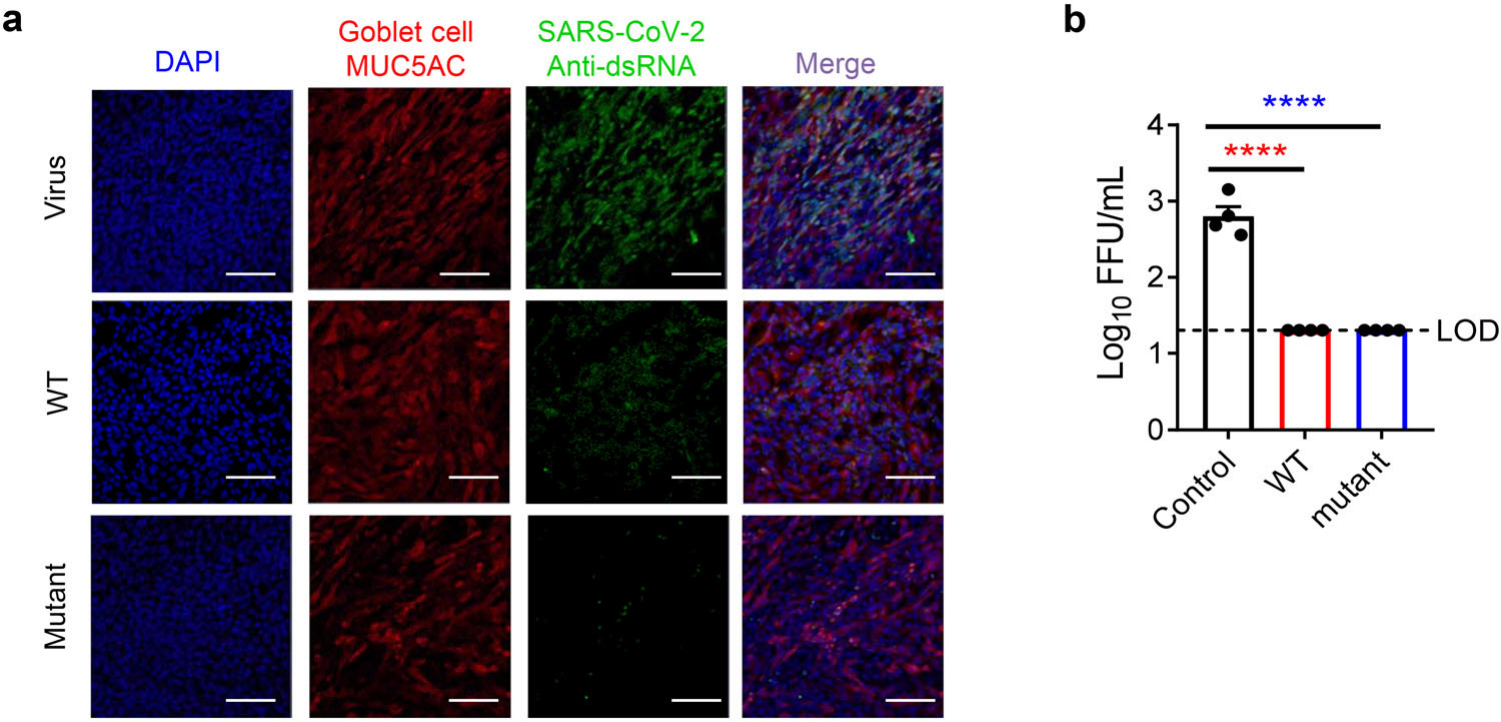
hACE2-Fc protected HBE from SARS-CoV-2 infection. **a** At 24 h post SARS-CoV-2 infection, HBE cells were fixed with 4% paraformaldehyde, followed by immunofluorescent staining. SARS-CoV-2 virus (green) and goblet cell (red) were stained with anti-dsRNA monoclonal antibody and anti-mucin 5AC monoclonal antibody, respectively. Nuclei were stained with DAPI (Blue). Scale bars (white), 100 µm. Magnification, ×20. **b** Cell culture supernatant was collected for virus titering. LOD, limit of detection. Data are representative of four independent experiments. *P ≤ 0.05, **P ≤ 0.01, ***P ≤ 0.001, ****P ≤ 0.0001.

Further, to test the protective effect of hACE2-Fc against SARS-CoV-2 in human primary cells, HBE cultures were generated^50,51^. HBE cells are commonly used for isolation and culture of respiratory viruses. Here, HBE cells were infected with SARS-CoV-2 virus in the presence of WT or mutant hACE2-Fc. Immunofluorescent staining of infected HBE cells was performed 24 h post infection. As shown in Fig. 3a, SARS-CoV-2 primarily infected goblet cells (MUC5AC-positive cells). Both the hACE2-Fc WT- and mutant-treated HBE cells showed significant reduction of SARS-CoV-2 infection. Deceased viral loads were observed in the apical wash (Fig. 3b), indicating hACE2-Fc treatment also efficiently inhibited SARS-CoV-2 infection in primary human epithelial cells.

### Prophylactic and therapeutic administration of hACE2-Fc protected SARS-CoV-2-infected mice

**Fig. 4:**
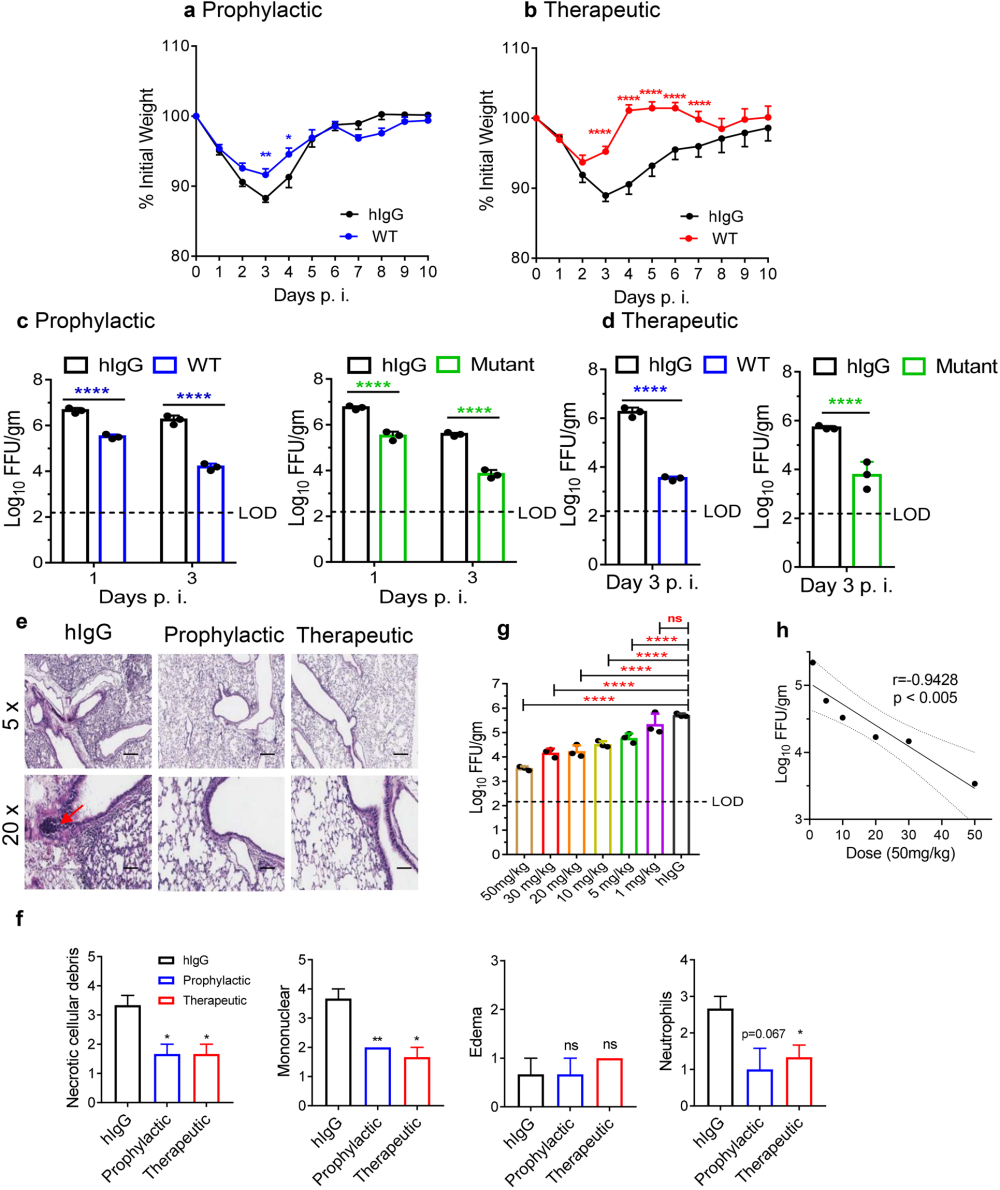
hACE2-Fc protected mice from SARS-CoV-2 infection. hACE2-Fc WT was administrated to Ad5-ACE2-transduced BALB/c mice (6–7 weeks old, female, average weight 18.69 ± 0.92 g) intraperitoneally at a dose of 50 mg/kg one day before (prophylactic) or after (therapeutic) SARS-CoV-2 infection (1 × 10^5^ PFU, intranasally). Equivalent human IgG was administrated as a negative control. **a, b** Weight loss was monitored for 10 days (*n* = 5 mice per group). **c, d** Lungs were harvested for viral titers at 1 dpi and 3 dpi for prophylactic group and 3 dpi for therapeutic group (*n* = 3 mice per group). LOD limit of detection, gm gram. **e** Lung tissues were harvested at 4 dpi. Sections were stained with H&E (*n* = 3 mice per group). Arrowhead indicates regions with focal infiltration of inflammatory cells. Representative pathological images were from three independent mice per group. Scale bars (black), 400 µm at 5× and 100 μm at 20×, respectively. **f** Summary of histology score (necrotic cellular debris, mononuclear, edema and neutrophils) at 4 dpi. Score criteria was performed as described in Materials and Methods. Multiple comparisons following 1-way ANOVA and Kruskal–Wallis test were performed for statistical analysis. Bonferroni’s correction was used to avoid inflation of experiment-wise Type I error. **g** hACE2-Fc WT was administered at 1 dpi (50 mg/kg, 30 mg/kg, 20 mg/kg, 10 mg/kg, 5 mg/kg, 1 mg/kg). Lungs were harvested for viral titers at 3 dpi (*n* = 3 mice per group). LOD limit of detection. Multiple comparisons following one-way ANOVA and Kruskal–Wallis test were performed for statistical analysis. Bonferroni’s correction was used to avoid inflation of experiment-wise Type I error. **h** Pearson’s correlation coefficient was used to assess the correlation between lung viral titer and hACE2-Fc administration dose. *P ≤ 0.05, **P ≤ 0.01, ***P ≤ 0.001, ****P ≤ 0.0001.

The prophylactic and therapeutic efficacies of WT and mutant hACE2-Fc were evaluated in vivo using Ad5-hACE2-transduced mice challenged with SARS-CoV-2^5^. Ad5-hACE2-transduced BALB/c mice were treated with WT or mutant hACE2-Fc at a dose of 50 mg/kg intraperitoneally (i.p.) 1 day before or 1 day after SARS-CoV-2 infection. As shown in Fig. 4a, b, mice in both prophylactic and therapeutic groups (*n* = 5 per group) showed significantly reduced weight losses after infection as compared to hIgG-treated control mice. Lungs (*n* = 3 per group) were harvested for virus titration at days 1 and 3 post infection (dpi) in the prophylactic group and at 3 dpi in the therapeutic group. In the prophylactic group, lung virus titers decreased 1 log at 1 dpi, and 2 logs at 3 dpi (Fig. 4c). Surprisingly, hACE2-Fc WT treatment showed a stronger protective effect in the therapeutic group, with reduced viral loads of approximately 3 logs in the lungs at 3 dpi. In the mutant hACE2-Fc-treated group, similar lung virus titer decreases were observed (Fig. 4d). Lung sections were obtained at 4 dpi. Compared with control mice, hACE2-Fc treatment prevented peribronchial lymphoid infiltration and bronchial epithelial cell damage (Fig. 4e, f). To explore the dose-dependent therapeutic effects of hACE2-Fc, Ad5-hACE2 -transduced mice were administrated i.p. with different doses of WT hACE2-Fc (50, 30, 20, 10, 5, and 1 mg/kg) 1 day post SARS-CoV-2 infection (*n* = 3 per group). Lungs were harvested for viral titers two days later by using Focus Forming Assay as described in Materials and methods. As shown in Fig. 4g, h, hACE2-Fc administration of doses higher than 5 mg/kg reduced viral loads in the lungs. The decreased viral loads in the lung correlated with increased amounts of hACE2-Fc, indicating that the therapeutic efficacy was dose-dependent.

### Pulmonary gene expression changes in response to SARS-CoV-2 infection and hACE2-Fc treatment

**Fig. 5:**
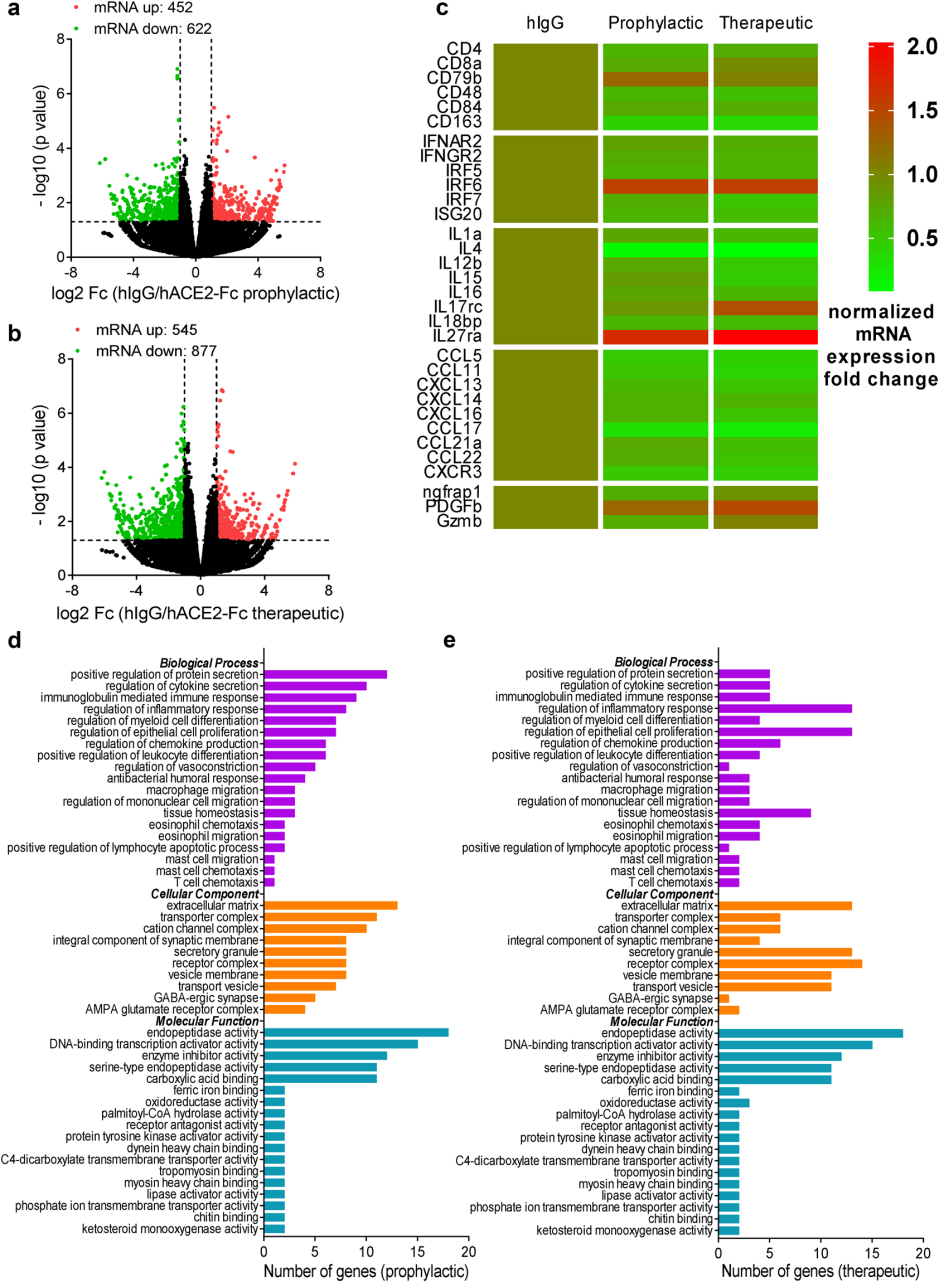
Pulmonary gene expression changes in response to SARS-CoV-2 infection and hACE2-Fc administration. **a, b** Volcano plot showing differential mRNA expression in the lungs of hACE2-Fc prophylactic and therapeutic administrated mice, as compared to hIgG administrated mice. The upregulated and downregulated genes are marked by red and green colors, respectively. **c** Representative normalized mRNA expression fold changes among prophylactic, therapeutic and hIgG control groups were shown. **d, e** The KEGG annotation results of genes with different expression levels in hACE2-Fc prophylactic and therapeutic administrated mice compared to hIgG administrated mice. The functional categories including biological processes, cellular component, and molecular function were shown with different colors.

To determine if hACE2-Fc treatment altered the lung inflammatory milieu, in addition to blocking viral entry, bulk RNA sequencing was performed using lung tissues harvested at 3 dpi from both prophylactic and therapeutic groups. Gene expression changes were determined as mRNA fold change relative to control group injected with human IgG (hIgG). In total, 1074 genes were differentially expressed, with 452 genes upregulated and 622 genes downregulated in the prophylactic group compared to the control group (Fig. 5a). Similarly, 545 genes were upregulated and 877 genes were downregulated in the therapeutic group (Fig. 5b). A heatmap of a representative 32 upregulated or downregulated genes shared by both the prophylactics and therapeutic groups sorted by DESeq2 software is shown in Fig. 5c. Compared to mice treated with control hIgG, T cell migration markers, CD4 and CD8 abundance were downregulated in hACE2-Fc-treated mice, while B cell migration abundance determined by CD79b transcript expression was upregulated. In addition, CD48, CD84, and CD163, mostly expressed on lymphocytes, monocytes, mast cells, macrophages, and NK cells were downregulated in hACE2-Fc treated mice. Compared to hIgG-treated mice, genes responsible for pro-inflammatory response, chemokine, transport, and metabolism were significantly downregulated in prophylactically and therapeutically treated mice (Fig. 5c), indicating that hACE2-Fc treatment could significantly reduce lung inflammation. The Kyoto Encyclopedia of Genes and Genomes (KEGG) annotation of genes with different expression levels in mice treated prophylactically and therapeutically with hACE2-Fc compared to hIgG is shown in Fig. 5d, e. In total 47-representative enriched KEGG functional categories were identified, and differential enrichment between the prophylactic and therapeutic groups was observed. Upregulated or downregulated genes were distributed in biological processes, cellular component, and molecular function pathways. There was no significant difference between prophylactically and therapeutically treated groups.

### hACE2-Fc has a relatively long half-life in vivo

**Fig. 6:**
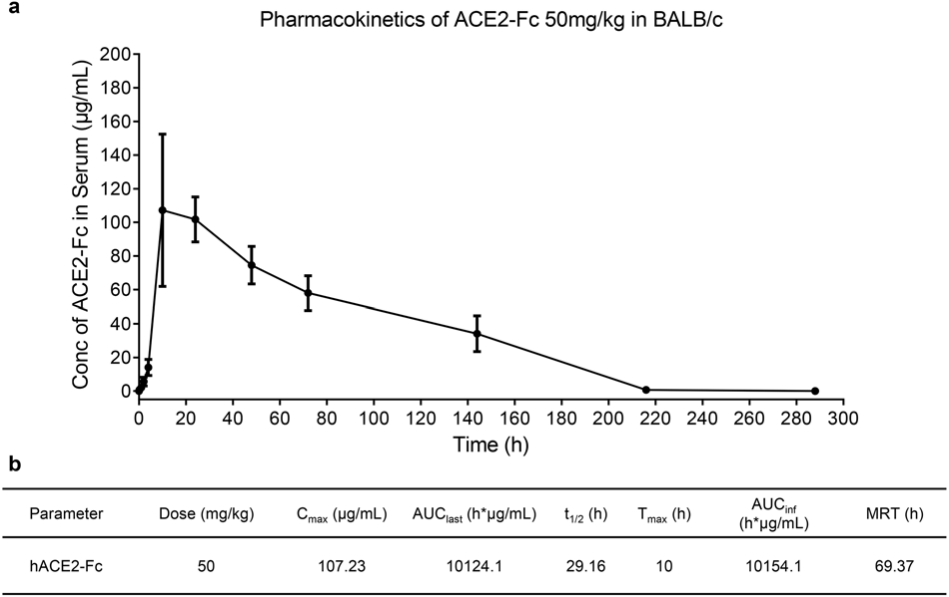
hACE2-Fc had prolonged half-life in vivo. **a** Serum samples were collected from mice (*n* = 12) at 0 min, 15 min, 1 h, 2 h, 4 h, 10 h, 24 h, 2 d, 3 d, 6 d, 9 d, 12 d, 16 d, and 22 d post administration (50 mg/kg). Concentration of hACE2-Fc in serum was determined by ELISA and analyzed by a standard curve. Date points are mean ± SD of six biological replicates. **b** Pharmacokinetic parameters of hACE2-Fc were obtained by WinNonlin 8.1 noncompartment model. AUC_last_ area under the curve, C_max_ maximum plasma concentration, T_max_ time to reach maximum plasma concentration, t_1/2_ terminal half-life, AUC_inf_ AUC from time zero to infinity, MRT mean residence time.

To determine the pharmacokinetics (PK) of hACE2-Fc in vivo, mice were treated with hACE2-Fc at a dose of 50 mg/kg. Sera were collected from mice at different timepoints after treatment and used for PK analysis. As showed in Fig. 6, the concentration of serum hACE2-Fc reached C_max_ (107.23 μg/mL) at 10 h and then slowly declined with a half-life (t_1/2_) of 29.16 h, which is much longer than human and mouse ACE2 (human: 10.0 h; mouse: 8.5 h)^52^. This slow decay could also explain why the protective efficacy of hACE2-Fc in SARS-CoV-2-infected mice after therapeutic administration was more potent than after prophylactic use since hACE2-Fc levels decreased by about 50% before viral infection in the prophylactic group (Fig. 4).

## Discussion

COVID-19 has spread to more than two hundred countries, areas, or territories. Multiple therapeutic and prevention strategies are being developed against SARS-CoV-2 infection, including vaccines^53–56^, repurposed drugs^7^, convalescent plasma^57,58^, and neutralizing antibodies^18–22^. Blocking the binding of SARS-CoV-2 spike protein and its cell receptor hACE2 at the early stage of virus infection will be an effective approach. hACE2 fused with human IgG-Fc region may be a promising choice for early COVID-19 treatment since hACE2-Fc has a prolonged half-life in vivo and can maintain a proper blood concentration for long-term therapeutic effects^35^. Structural analysis indicated that hACE2 interacts with RBD of SARS-CoV-2 in the form of dimer, thus fusion of hACE2 with Fc may stabilize the structure of ACE2 for RBD binding^59^. Not only for SARS-CoV-2, but this approach might also work for other emerging virus infections. Once the cell receptor for the emerging virus is identified, the cellular receptor fused with Fc could be generated and expressed as a therapeutic drug rapidly.

Here, we demonstrated that hACE2-Fc, a recombinant hACE2 extracellular domain fused with Fc region, had a potent effect to neutralize SARS-CoV-2 authentic virus as efficiently as neutralizing antibodies. Importantly, we found that hACE2-Fc can neutralize most of VOCs (Alpha strain, Beta strain, and Delta strain) and a Variant of Interest (Kappa strain), which suggests the potential neutralization capacity of hACE2-Fc to more emerging SARS-CoV-2 variants with inscrutable public health threat. In addition, our results indicated that hACE2-Fc has broadly neutralizing activities against both HCoV-NL63 and SARS-CoV in vitro, although the antiviral activity of hACE2-Fc against SARS-CoV and HCoV-NL63 in vivo remained to be determined further. Besides, hACE2-Fc did not promote SARS-CoV-2 infection even at very low concentration. A study by Yeung et al.^60^ reported that soluble ACE2 may promote SARS-CoV-2 entry by forming an sACE2-S complex to enter cells through receptor-mediated endocytosis via the AT1 or forming an sACE2-S-vasopressin complex to enter cells via AVPR1B. We assumed that fusing with Fc region did not alter the binding of ACE2 to SARS-CoV-2 but obstruct the forming of complexes.

Moreover, hACE2-Fc can not only inhibit SARS-CoV-2 entry, but also inhibit SARS-CoV-2 spike-mediated cell–cell fusion, highlighting one more potential beneficial effect of hACE2-Fc in addition to blocking entry. The capacity of cell–cell fusion inhibition of hACE2-Fc was not as potent as blocking entry, indicating that blocking entry is essential for hACE2-Fc protective effect, while inhibiting cell–cell fusion may serve as an auxiliary capacity of hACE2-Fc. In addition, multiple SARS-CoV-2 variants are circulating globally, including B.1.1.7, B.1.351, B.1.617.2, and B.1.617.1. Some variants may be associated with an increased risk of transmission and death compared with the initial strain^40,61^. hACE2-Fc covers the entire binding site of RBD and ACE2, which greatly decreases the possibility of escaping by SARS-CoV-2 variants as well as diminishing the generation of escape mutants during patient treatment. It is potentially useful for the whole course of COVID-19 pandemic.

Further, by using an Ad5-hACE2 transduction murine model for COVID-19^5^, hACE2-Fc showed potent protective effects in SARS-CoV-2-infected mice. Prophylactic and therapeutic hACE2-Fc treatment strongly inhibited SARS-CoV-2 replication in lungs and prevented SARS-CoV-2-induced lung lesions. Most importantly, the protective effect of hACE2-Fc was dose-dependent and correlated with the result of PK in vivo, since the protective effect in therapeutic group was stronger than that in the prophylactic group. Recently, a case report provided encouraging data on the use of recombinant soluble hACE2 to treat a severe COVID-19 patient^62^. The data documented multiple significant improvement of the patient’s clinical complications after treatment, including decreased SARS-CoV-2 viral load and reduced inflammatory response. Notably, the use of hACE2 did not impede the generation of neutralizing antibodies^63^. Together, both our animal experiments and the case report indicate that recombinant soluble hACE2 could be a promising treatment for severe COVID-19.

Besides, two relevant studies have reported the half-life of hACE2-Fc in mice^30,64^. There are multiple known factors that may affect the PK value observed in hACE2-Fc test, including the sequence of Fc region of fusion protein, manufacture process (particularly the cell line used for expression, which significantly affects the post translational modification and in vivo PK) and animal model, etc. The half-life of hACE2-Fc (29.16 h) in mice is longer than that of soluble recombinant ACE2, which was 8.68 ± 0.79 h for soluble mouse ACE2 in mice (intraperitoneal administration) and 10 h for soluble hACE2 in healthy subjects (intravenous administration)^35,52,65^. The Fc region of hACE2-Fc may contribute to the in vivo retention time for making long-lasting effect for SARS-CoV-2 inhibition.

Mutations that inactivate ACE2’s catalytic activity did not reduce its protective effect in vivo against SARS-CoV-2. In this study, the catalytically inactive hACE2-Fc mutant also exhibited similar protective effect in vitro and in vivo. Engineering an optimal ACE2 protein with potent neutralizing activity against SARS-CoV-2 and prolonged half-life^20,33^ is the major goal for ACE2-derived therapeutic drug development for COVID-19. In summary, the data presented here demonstrated the efficacy of hACE2-Fc in prophylactic and therapeutic treatment regimens in vivo, which is a potentially excellent candidate for COVID-19 therapy.

## Materials and methods

### Expression of hACE2-Fc and mutant hACE2-Fc

Fc domain-Fusion could prolong the half-life and improve the solubility and stability of protein of interest^35^. Based on the amino acid sequence of hACE2 deposited in GenBank, optimized ECDs of hACE2 sequences were ligated to the DNA sequence of Fc segment of human IgG1 and inserted into an expression plasmid. Recombinant fusion proteins hACE2-Fc was expressed by Expi293 cells (Thermo) and purified by Protein A magnetic beads. In addition, an inactivated hACE2 (a mutated form, H374N and H378N mutations) also was generated as described above and named as hACE2-Fc mutant. Purified proteins were analyzed by SDS-PAGE, and protein concentration was determined by BCA protein assay.

### Kinetics and affinity determination by SPR assay

Binding affinity and kinetics of hACE2-Fc and hACE2-Fc mutant were performed by SPR method using Biacore 8 K (GE healthcare). The immobilization of SARS-CoV-2 RBD and SARS-CoV-2 spike ECD proteins (extracellular domains, 16–1213 aa) were performed under 25 °C and HBS-EP was used as the running buffer. The sensor chip surface of flow cells 1 and 2 was activated by freshly mixed 50 mmol/L N-Hydroxysuccinimide (NHS) and 200 mmol/L 1-ethyl-3-(3-dimethylaminopropyl) carbodiimide hydrochloride (EDC) for 200 s (10 µL/min). Then, RBD or spike ECD proteins diluted in 10 mmol/L NaAC (pH 4.5) was injected into the flow cell 2 to achieve conjugation of appropriate Response Unit, respectively, while flow cell 1 was set as blank. After the amine coupling reaction, the remaining active coupling sites on chip surface were blocked with 200 s injection of 1 mol/L ethanolamine hydrochloride. Then, diluted hACE2-Fc was injected over the surface as association phase, followed by injecting running buffer as dissociation phase.

### ELISA assay

ELISA was used to determine the EC_50_s of hACE2 against spike or RBD proteins. SARS-CoV-2 RBD and SARS-CoV-2 spike ECD proteins were coated with 96-well plate (0.5 μg/mL) at 4 °C overnight. After blocked for 2 h by 10% fetal bovine serum at 37 °C, serially diluted hACE2-Fc was added and incubated for 2 h. After washing, HRP-conjugated mouse anti-human IgG (H + L) antibody (Jackson ImmunoResearch) was used as secondary antibody. Finally, TMB as the substrate solution was added into plates after thorough washing. OD_450_ value was obtained using microplate reader (BioTek instruments, Inc).

### Neutralization test based on SARS-CoV, SARS-CoV-2, and HCoV-NL63 pseudotyped virus (pp)

SARS-CoV pp and SARS-CoV-2 pp were generated by lentiviral pseudotyped system as described previously^66,67^. In brief, HEK293 cells were co-transfected with reporter plasmid plenti-GFP-luc, packaging plasmid psPAX2, and coronavirus spike plasmid of interest (pcDNA3.1-SARS-CoV-S, pcDNA3.1-SARS-CoV-2-S, pcDNA3.1-SARS-CoV-2-S-D614G, pcDNA3.1-SARS-CoV-2-S-V367F, pcDNA3.1-B.1.1.7-S, pcDNA3.1-B.1.351-S, pcDNA3.1-B.1.351-S, pcDNA3.1-B.1.617.1-S, and pcDNA3.1-B.1.617.2-S) by lipofectamine 2000 (Thermo). Viral supernatant was harvested and used for neutralization test 48 h post transfection. HCoV-NL63 pp was generated by vesicular stomatitis virus (VSV) pseudotyped system^33^. HCoV-NL63 spike plasmid (pcDNA3.1-HCoV-NL63-S) was transfected into 293 T cells, 24 h later transduced target cells were infected with rVSV-ΔG-luc, and viral supernatant was harvested 24 h after transduction. Lenti-X p24 Rapid Titer Kit was used for HIV-1-based lentivirus titering (Takara Bio, Cat. No. 632200). Briefly, anti-HIV-1 p24 capture antibody was coated in 96-well ELISA plate, and serially diluted test samples and standards were then added. Specifically-bound p24 was detected in a typical sandwich ELISA format using a biotinylated anti-p24 secondary antibody, a streptavidin-HRP conjugate, and a color producing substrate. The pesudotyped virus of SARS-CoV-2 WT and its variants were normalized based on the result of p24 ELISA kit and identical multiplicity of infection (MOI) was used for infection. SARS-CoV-pp and SARS-CoV-2-pp neutralization assay were performed on ACE2-overexpressing HEK293 cell (HEK293-ACE2 cell) in a 96-well microplate^68^, while HCoV-NL63 pp neutralization assay was conducted on Huh7 cell as previously described^33^. Briefly, 50 μL of serially diluted fusion proteins were added to an equal volume of the coronavirus pp and incubated for 60 min at 37 °C, then 100 μL of mixture were added to 96-well plate which was seeded with target cells (HEK293-ACE2 or Huh7 cells) at an appropriate density 18 h before infection. Cells were incubated for 40 h at 37 °C and were then lysed. Luciferase activity was measured using steady-Glo luciferase assay system (Promega).

### Focus forming assay (FFA) for SARS-CoV-2 quantification

All SARS-CoV-2 infection experiments were performed in a biosafety level-3 laboratory. Vero E6 cells were seeded onto 96-well plates overnight and grown into confluent monolayers. Fifty microliters of 10-fold-diluted SARS-CoV-2 stock or supernatant of lung homogenate was added into 96-well plate and adsorbed at 37 °C for 1 h with rocking every 10 min. Then the virus or supernatant of lung homogenate were removed and covered with 100 μL Minimum Essential Medium (MEM) containing 1.2% Carboxymethylcellulose (1.2% CMC). After 24 h post infection, the overlay was discarded and the cell monolayer was fixed with 4% paraformaldehyde solution for 2 h at room temperature (RT). After permeabilized with 0.2% Triton X-100 for 20 min at RT, the plates were sequentially stained with cross-reactive rabbit anti-SARS-CoV-N IgG (Sino Biological Inc) as the primary antibody and HRP-conjugated goat anti-rabbit IgG(H + L) (Jackson ImmunoResearch) as the secondary antibody in 37 °C for 1 h, respectively. The reactions were developed with KPL TrueBlue Peroxidase substrates. The numbers of SARS-CoV-2 foci were calculated using CTL ImmunoSpot S6 Ultra reader (Cellular Technology Ltd).

### Focus reduction neutralization test (FRNT) of SARS-CoV-2

FRNT assay was used for the evaluation of hACE2-Fc neutralization effect. Vero E6 cells were seeded into a 96-well plate one day before infection. The next day, serially diluted hACE2-Fc proteins and SARS-CoV-2 (80–100 FFU, Focus forming unit) were combined in DMEM (Dulbecco’s Modified Eagle Medium) containing 2% Fetal Bovine Serum and incubated at 37 °C for 1 h. Then, fifty microliters mixtures were added into 96-well plate seeded with Vero E6 cells and incubated in 37 °C for 1 h. The following steps were the same as FFA method mentioned above. The IC_50_s is determined by 50% focus reduction neutralization test titers (FRNT_50_) which was used for evaluation of the potency of hACE2-Fc in inhibiting SARS-CoV-2 replication.

### Cell–cell fusion inhibition assay

Cell–cell fusion assay was conducted as previously described^68^. Briefly, plasmids encoding SARS-CoV-2 spike protein and pEGFP were co-transfected into HEK293T cell. Forty-eight hours post transfection, effector cells (transfected HEK293T cells) were harvested and incubated with serially diluted hACE2-Fc WT or mutant at 37 °C for 1 h. Effector cells and target cells (Huh7 cells) were mixed 1:1 with a total cell density of 5 × 10^4^ in 96-well plates. After 24 h, plates were checked for the forming of syncytia under a fluorescence microscope (EVOS digital inverted microscope, Invitrogen).

### SARS-CoV-2 escape mutant assay

A strain of SARS-CoV-2 (isolated SARS-CoV-2/human/CHN/IQTC01/2020, GenBank: MT123290.1) isolated from an infected patient was passaged in Vero E6 cell and Calu-3 cell cultures supplemented with increasing concentration of serially diluted hACE2-Fc WT, while the same SARS-CoV-2 strain passaged without the existence of hACE2-Fc was used as a control. Passages were conducted with infection MOI ranging from 0.01 to 0.05 and hACE2-Fc concentration ranging from 0.4× to 4 × IC_50_ (determined by SARS-CoV-2 authentic virus neutralization assay in Vero E6 cells). Vero E6 cell supernatant was harvested at 72 h post infection for next-round passage with higher concentration of hACE2-Fc when the cytopathic effect of cells reached 50%–60%, while Calu-3 cell supernatant was harvested at 48 h post infection. To identify changes in RBD-associated with hACE2-Fc escape, viral RNA was extracted for next generation sequencing to determine the mutant sites. Sequences were analyzed by using CLC Genomics Workbench version 11 (Qiagen) and MEGA 5.10.

### SARS-CoV-2 infection and immunofluorescent staining of hACE2-Fc-treated HBE

HBE cells were obtained by fiberoptic bronchoscopy and brushing of airway walls from healthy donors and cultured at the air–liquid interface (ALI) on transwells as described^50,51^. Cells were maintained and expanded (one passage) in T75 flasks in human airway epithelial cell expansion medium (PneumaCult-Ex Plus Medium Kit, Stemcell, Catalog #05040) at 37 °C in a 5% CO_2_ incubator. At 80% confluence, cells were detached with 0.05% trypsin-EDTA (Gibco) and seeded on membrane supports (12 mm Transwell culture inserts, 0.4 µm pore size, Costar) coated with 0.05 mg collagen from calf skin (Sigma–Aldrich) in airway epithelial cell growth medium. HBE cells were cultured for two days until they reached complete confluence. The apical medium was then removed and the basal medium was replaced by medium for human airway epithelial cells cultured at the air–liquid interface (PneumaCult-ALI Medium Kit, Stemcell, Catalog #05001). Cultures were maintained under air–liquid interface conditions by changing the medium in the basal filter chamber every 48 h. For SARS-CoV-2 infection, epithelial cells were cultured in differentiation medium containing SARS-CoV-2 at 37 °C in a 5% CO_2_ incubator from day 14 to day 15 before collection for analysis. Well-differentiated HBE cells in permeable transwells reach a density of 3.5–4.0 × 10^5^ cells per well before infection. Diluted hACE2-Fc WT/mutant (at a concentration of 20× or 2 × IC_50_ in authentic SARS-CoV-2-infected Vero E6 cell neutralization assay) and SARS-CoV-2 (MOI = 0.2) were combined in HBE cell culture medium and incubated at 37 °C for 1 h. After rinsing the apical and basolateral surfaces of transwells with Dulbecco’s phosphate-buffered saline (DPBS), five hundred microliters mixtures were added to each of the apical and basolateral surfaces of HBE cells in transwells at 37 °C for 1 h. At 24 h post infection, cell culture was collected for virus titering, HBE cells were fixed with 4% paraformaldehyde solution for 2 h at RT. Membranes were incubated with 0.2% Triton X-100 for 10 min, then stained with mouse anti-dsRNA monoclonal antibody J2 (SCICONS, 10010200) and rabbit anti-MUC5AC monoclonal antibody (Abcam, ab198294) as primary antibodies at 4 °C overnight. After staining with Alex Fluor 488 goat anti-mouse IgG and Alex Fluor 568 goat anti-rabbit IgG secondary antibodies, and incubating with nuclear staining dye (DAPI, Sigma–Aldrich LLC, D9542) in dark, membranes were cut out of the well and mounted on a slide for imaging (LSM 880 confocal microscope, Zeiss).

### Ad5-hACE2 transduction, hACE2-Fc administration, and SARS-CoV-2 infection of BALB/c mice

Ad5-hACE2-sensitized mice are useful for evaluation of antiviral therapies as we previously described^5^. Here we adopted this animal model for evaluation of hACE2-Fc prophylactic and therapeutic efficacy against SARS-CoV-2 infection in vivo. Briefly, specific pathogen-free 5–6-week-old female BALB/c mice (~15 g) were lightly anesthetized with isoflurane and transduced with 2.5 × 10^8^ FFU of Ad5-hACE2 5 days before intranasal challenge with 1 × 10^5^ FFU of SARS-CoV-2. For prophylactic group, mice were injected with 50 mg/kg (750 μg each) hACE2-Fc i.p. 1 day before infection. For therapeutic group, mice were treated with hACE2 one d after infection i.p. Mice were monitored daily for weight loss. Lungs were removed into PBS at indicated timepoints and homogenized. Virus titers of clarified supernatants were assayed in Vero E6 cells and expressed as FFU per gram of tissue. All work with SARS-CoV-2 was conducted in the Guangzhou Customs District Technology Center Biosafety Level 3 (BSL-3) Laboratory.

### Histology analysis

Four days after infection, mice were anesthetized and transcardially perfused with PBS followed by zinc formalin. Lungs were removed, fixed in zinc formalin, paraffin-embedded, and sectioned at 4 μm thickness. Sections were stained with hematoxylin/eosin for histological analysis. Photomicropraphs were taken using PRECICE 500B (UNIC TECHNOLOGIES, INC). Edema, necrotic cellular debris and mononuclear were scored according to the following criteria: 0—none; 1—uncommon detection in < 5% lung fields; 2—detectable in up to 33% of lung fields; 3—detectable in up to 33%–66% of lung fields; 4—detectable in > 66% of lung fields. Neutrophil infiltration was scored according to the following criteria: 0—within normal limits; 1—scattered PMNs sequestered in septa; 2—#1 plus solitary PMNs extravasate in airspaces; 3—#2 plus small aggregates in vessel and airspaces^5^.

### RNA sequencing and data analysis

Total RNA was extracted from infected mice lung using Trizol reagent (invitrogen). Ribosomal RNA was removed using EpicentreRibo-Zero rRNA Removal Kit (illumina, USA). According to previous reports^5^, the sequencing procedures were performed as follows. In brief, purified RNAs were fragmented to approximately 200 bp, and collected RNAs were subjected to cDNA synthesizing. The libraries were paired-end sequenced (PE150, 2 × 150 bp) using IlluminaHiSeq 3000 platform. Clean data were aligned to the mouse reference genome (mm10) via software HISAT2^69^. HTSeq v0. 6.0 was used to count the reads numbers mapped to each gene^70^. The whole-samples expression levels were presented as Transcripts Per Kilobase of exon model per Million mapped reads (TPM), which is the most frequently reported RNA-seq gene expression values. The statistically significant DE genes were obtained by an adjusted *P* value threshold of <0.05 and log2(fold change) > 1 using the DEGseq software^71^. Finally, a hierarchical clustering analysis was performed using the R language package gplots according to the TPM values of differential genes in different groups. And colors represent different clustering information, such as the similar expression pattern in the same group, including similar functions or participating in the same biological progress^72^. All the basic data series were submitted to NCBI SRA with accession number SRP286817 (BioProject: PRJNA667999).

### Pharmacokinetic properties of hACE2-Fc in mice

WT specific pathogen-free BALB/c mice were injected i.p. with 50 mg/kg (750 μg each) hACE2-Fc. Serum samples were collected from mice (*n* = 12, totally) at 0 min, 15 min, 1 h, 2 h, 4 h, 10 h, 24 h, 2 d, 3 d, 6 d, 9 d, 12 d, 16 d, and 22 d post administration. The concentration of hACE2-Fc in serum was analyzed by detecting IgG-Fc using ELISA. In brief, 96-well plate was coated with SARS-CoV-2 spike protein. hACE2-Fc was determined by HRP-conjugated mouse anti-human IgG-Fc antibody and TMB. Individual PK parameters were evaluated using a noncompartmental analysis (NCA) method with WinNonlin (Version 8.1). The following parameters were calculated or determined: area under the curve (AUC), maximum plasma concentration (C_max_), time to reach maximum plasma concentration (T_max_), terminal half-life (t_1/2_), AUC from time zero to infinity (AUC_inf_), and mean residence time (MRT).

### Accession numbers

The RNA-seq data supporting the finding in this study were deposited to the NCBI SRA with accession number SRP286817 (BioProject: PRJNA667999).

### Statistical analysis

Statistical analysis was performed using Graphpad Prism software, version 8.00. A Student’s *t* -test was used to analyze differences in mean values between groups. A log (inhibitor) vs response—Variable slope (four parameters) test was used for IC_50_ determination. Liner regression (pearson analysis) was used for correlation analysis. Multiple comparisons following one-way ANOVA and Kruskal–Wallis test were performed for statistical analysis. Bonferroni’s correction was used to avoid inflation of experiment-wise Type I error. *P*-values < 0.05 were statistically significant. (*P ≤ 0.05, **P ≤ 0.01, ***P ≤ 0.001, ****P ≤ 0.0001). All values are depicted as mean ± SEM.
